# Briarenolides F and G, New Briarane Diterpenoids from a *Briareum* sp. Octocoral

**DOI:** 10.3390/md10051156

**Published:** 2012-05-23

**Authors:** Pei-Han Hong, Yin-Di Su, Jui-Hsin Su, Yung-Husan Chen, Tsong-Long Hwang, Ching-Feng Weng, Chia-Hung Lee, Zhi-Hong Wen, Jyh-Horng Sheu, Nai-Cheng Lin, Yueh-Hsiung Kuo, Ping-Jyun Sung

**Affiliations:** 1 Graduate Institute of Marine Biotechnology, National Dong Hwa University, Pingtung 944, Taiwan; Email: peihan520@yahoo.com.tw (P.-H.H.); x2219@nmmba.gov.tw (J.-H.S.); cfweng@mail.ndhu.edu.tw (C.-F.W.); chlee016@mail.ndhu.edu.tw (C.-H.L.); 2 National Museum of Marine Biology and Aquarium, Pingtung 944, Taiwan; Email: gobetter04@yahoo.com.tw (Y.-D.S.); tony_chen72001@yahoo.com.tw (Y.-H.C.); lnc7222@hotmail.com (N.-C.L.); 3 Department of Marine Biotechnology and Resources and Division of Marine Biotechnology, Asia-Pacific Ocean Research Center, National Sun Yat-sen University, Kaohsiung 804, Taiwan; Email: wzh@mail.nsysu.edu.tw (Z.-H.W.); sheu@mail.nsysu.edu.tw (J.-H.S.); 4 Graduate Institute of Natural Products, Chang Gung University, Taoyuan 333, Taiwan; Email: htl@mail.cgu.edu.tw; 5 Department of Life Science and Institute of Biotechnology, National Dong Hwa University, Hualien 974, Taiwan; 6 Tsuzuki Institute for Traditional Medicine, China Medical University, Taichung 404, Taiwan

**Keywords:** briarenolide, briarane, *Briareum*, superoxide anion

## Abstract

Two new briarane diterpenoids, briarenolides, F (**1**) and G (**2**), were isolated from an octocoral identified as *Briareum* sp. The structures of briaranes **1** and **2** were established by spectroscopic methods and by comparison of the spectroscopic data with those of known briarane analogues. Briarenolide F was proven to be the first 6-hydroperoxybriarane derivative and this compound displayed a significant inhibitory effect on the generation of superoxide anion by human neutrophils.

## 1. Introduction

**Figure 1:**
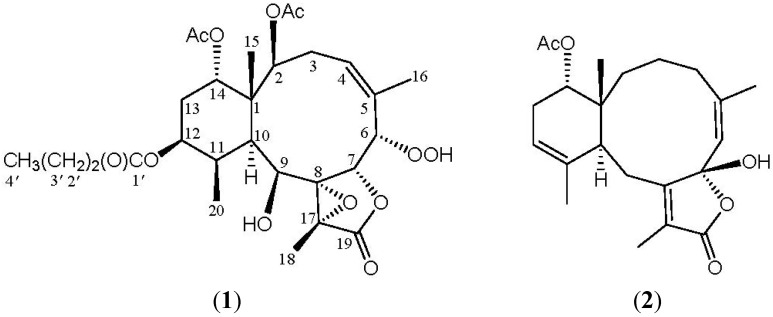
The structures of briarenolides F (**1**) and G (**2**).

Among the diterpenoids isolated from octocorals, the briarane-type metabolites (3,8-cyclized cembranes) are a major group of compounds [1,2,3]. The compounds of this type were suggested to be of marine origin and the octocorals belonging to the genus *Briareum* have been proven to be the most important source of briarane-type compounds [4,5,6,7]. In previous studies, a series of interesting terpenoid derivatives, including briarane [8,9,10,11,12,13,14,15,16,17,18,19,20,21,22,23,24,25,26,27,28,29,30,31,32,33,34,35], cembrane [36] and carotenoid [37], had been isolated from the octocorals belonging to the genus *Briareum* that were distributed in the waters off Taiwan, at the intersection point of the Kuroshio current and the South China Sea surface current. In a continuation of our search for new substances from the Formosan marine invertebrates, the chemical constituents of a specimen octocoral identified as *Briareum* sp. (Briareidae) were studied. A fraction of its organic extract (fraction H, see Experimental Section) displayed inhibitory effects on the generation of superoxide anion (inhibition rate 36.8%) and the release of elastase (inhibition rate 90.3%) at a concentration of 10 μg/mL. We further isolated two new briarane-type diterpenoids, briarenolides, F (**1**) and G (**2**) (Figure 1), from the octocoral *Briareum* sp. In this paper, we report the isolation, structure determination and bioactivity of briaranes **1** and **2**.

## 2. Results and Discussion

Briarenolide F (**1**) was isolated as a white powder. The molecular formula of **1** was established as C_28_H_40_O_12_ (nine degrees of unsaturation) from a sodium adduct at *m/z* 591 in the ESIMS spectrum and further supported by HRESIMS (C_28_H_40_O_12_Na, *m/z* 591.2420, alculated 591.2417). The IR spectrum of **1** showed bands at 3498, 1789 and 1743 cm^−1^, consistent with the presence of hydroxy, γ-lactone and ester carbonyl groups. The ^13^C NMR and DEPT spectra of **1** showed that this compound had 28 carbons (Table 1), including seven methyls, four sp^3^ methylenes, eight sp^3^ methines, three sp^3^ quaternary carbons, an sp^2^ methine and five sp^2^ quaternary carbons. From the ^1^H and ^13^C NMR spectra (Table 1), **1** was found to possess two acetoxy groups (δ_H_ 1.99, 2.01, each 3H × s; δ_C_ 170.6, 2 × qC; 21.3, 2 × CH_3_), an *n*-butyrate group (δ_H_ 0.94, 3H, t, *J* = 7.2 Hz; 1.63, 2H, sext, *J* = 7.2 Hz; 2.27, 2H, t, *J* = 7.2 Hz; δ_C_ 13.7, CH_3_; 18.4, CH_2_; 36.3, CH_2_; 173.1, qC), a γ-lactone moiety (δ_C_ 171.0, qC-19) and a trisubstituted olefin (δ_H_ 5.65, 1H, br d, *J* = 13.6 Hz, H-4; δ_C _130.3, CH-4; 128.8, qC-5). The presence of a tetrasubstituted epoxide containing a methyl substituent was established from the signals of two quaternary oxygenated carbons at δ_C_ 68.8 (qC-8) and 58.4 (qC-17) and further confirmed by the proton signal of a methyl singlet at δ_H_ 1.49 (3H, s, H_3_-18). Thus, from the above NMR data, five degrees of unsaturation were accounted for and **1** was identified as a tetracyclic compound. 

**Table 1:** ^1^H (400 MHz, CDCl_3_) and ^13^C (100 MHz, CDCl_3_) NMR data, ^1^H–^1^H COSY and HMBC correlations for briarane **1**.

C/H	δ (*J* in Hz)	δ_C,_ Mult.	^1^H–^1^H COSY	HMBC (H→C)
1		44.9, qC		
2	5.22 d (8.0)	75.4, CH	H_2_-3	C-1, -4, -10, -15, acetate carbonyl
3α	1.89 m	33.3, CH_2_	H-2, H-3β, H-4	N.O.
β	3.81 dd (17.2, 13.6)		H-2, H-3α, H-4	C-4, -5
4	5.65 br d (13.6)	130.3, CH	H_2_-3, H_3_-16	N.O.
5		128.8, qC		
6	4.65 d (2.4)	84.9, CH	H-7	C-4, -5, -7, -8, -16
7	5.33 d (2.4)	77.2, CH	H-6	C-5, -6
8		68.8, qC		
9	4.59 d (4.8)	75.1, CH	OH-9	C-1, -8, -10, -11, -17
10	2.06 d (4.0)	39.4, CH	H-11	C-1, -8, -9, -15, -20
11	2.28 m	40.2, CH	H-10, H-12, H_3_-20	C-1, -10, -12
12	4.98 ddd (12.4, 5.2, 5.2)	70.7, CH	H-11, H_2_-13	C-20, *n*-butyrate carbonyl
13	1.87–1.97 m	26.5, CH_2_	H-12, H-14	C-12
14	4.88 dd (3.2, 2.4)	74.9, CH	H_2_-13	N.O.
15	1.37 s	15.9, CH_3_		C-1, -2, -10, -14
16	1.77 br s	25.4, CH_3_	H-4	C-4, -5, -6
17		58.4, qC		
18	1.49 s	8.8, CH_3_		C-8, -17, -19
19		171.0, qC		
20	1.22 d (7.2)	10.6, CH_3_	H-11	C-10, -11, -12
2-OAc	1.99 s	170.6, qC21.3, CH_3_		Acetate carbonyl
6-OOH	8.71 br s			N.O.
9-OH	2.95 d (4.8)		H-9	N.O.
12-OC(O)Pr		173.1, qC	**	**
	2.27 t (7.2)	36.3, CH_2_	H_2_-3 *'* ** **	C-1 *'* , -3 *'* , -4 *'*
	1.63 sext (7.2)	18.4, CH_2_	** H_2_-2 *'* , H_3_-4 *'* **	C-1 *'* , -2 *'* , -4 *'*
	0.94 t (7.2)	13.7, CH_3_	H_2_-3 *'*	C-2 *'* , -3 *'*
14-OAc		170.6, qC		
	2.01 s	21.3, CH_3_		Acetate carbonyl

N.O. = Not observed.

**Figure 2:**
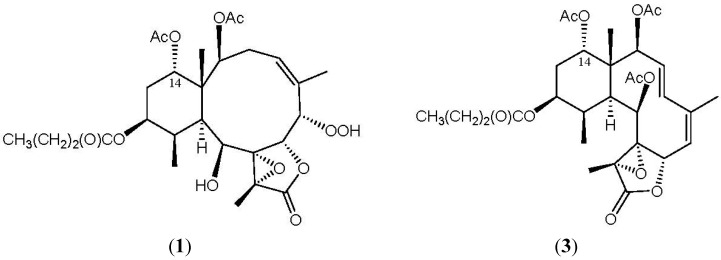
The structures of briarenolide F (**1**) and excavatolide F (**3**).

^1^H–^1^H couplings in the COSY spectrum of **1** enabled identification of the C-2/-3/-4, C-6/-7, C-10/-11/-12/-13/-14, C-4/-16 (by allylic coupling) and C-11/-20 units (Table 1), which were assembled with the assistance of an HMBC experiment. The HMBC correlations between protons and quaternary carbons of **1**, such as H-2, H-9, H-10, H-11, H_3_-15/C-1; H-3β, H-6, H-7, H_3_-16/C-5; H-6, H-9, H-10, H_3_-18/C-8; H-9, H_3_-18/C-17; and H_3_-18/C-19, permitted the elucidation of the carbon skeleton (Table 1). The vinyl methyl at C-5 was confirmed by the allylic coupling between H-4/H_3_-16 in the ^1^H–^1^H COSY spectrum and by the HMBC correlations between H_3_-16/C-4, -5, -6 and H-6/C-16. The ring junction C-15 methyl group was positioned at C-1 from the HMBC correlations between H_3_-15/C-1, -2, -10, -14; H-2/C-15; and H-10/C-15. In addition, the carbon signal at δ_C_ 173.1 (qC) was correlated with the signal of the methylene protons at δ_H_ 2.27 in the HMBC spectrum and was consequently assigned as the carbon atom of the *n*-butyrate carbonyl. Additionally, the *n*-butyrate positioned at C-12 was confirmed by the connectivity between H-12 (δ_H_ 4.98) and the carbonyl carbon (δ_C_ 173.1, qC) of the *n*-butyrate. Furthermore, an acetate ester at C-2 was established by a correlation between H-2 (δ_H_ 5.22) and the acetate carbonyl (δ_C_ 170.6, qC) observed in the HMBC spectrum of **1**. The presence of a hydroxy group at C-9 was deduced from the ^1^H–^1^H COSY correlation between a hydroxy proton (δ_H_ 2.95) and H-9 (δ_H_ 4.59). The presence of a hydroperoxy group in **1** was supported by a hydroperoxy proton signal at δ_H_ 8.71 as a broad signlet [22,32,38]. Due to absence of HMBC correlations for H-14 (δ_H_ 4.88) and the hydroperoxy proton (δ_H_ 8.71), the positions for the remaining acetoxy and hydroperoxy groups could not be determined by this method. By comparison the ^1^H and ^13^C NMR data of C-14 oxymethine for **1** (δ_H_ 4.88; δ_C_ 74.9) with those of a known briarane analogue, excavatolide F (**3**) (δ_H_ 4.94; δ_C_ 74.1) (Figure 2) [10], which possesses a similar cyclohexane moiety as that of **1**, the remaining acetoxy group in **1** was placed at C-14. Thus, the hydroperoxy group is positioned at C-6, an oxymethine at δ_C_ 84.9 (CH), by analysis of the ^1^H–^1^H COSY correlations and characteristic NMR signal analysis. 

**Figure 3:**
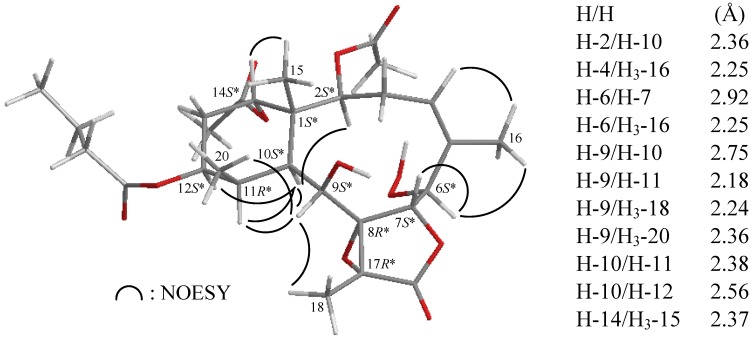
The stereoview of **1** (generated from computer modeling) and the calculated distances (Å) between selected protons with key NOESY correlations.

In all naturally-occurring briaranes, H-10 is *trans* to the C-15 methyl group, and these two groups are assigned as α- and β-oriented in most briarane derivatives [4,5,6,7]. The relative configuration of **1** was elucidated from the interactions observed in a NOESY experiment and was found to be compatible with that of **1** offered by computer modeling (Figure 3) [39] and that obtained from vicinal proton coupling constant analysis. In the NOESY experiment of **1**, the correlations of H-10 with H-2, H-9, H-11 and H-12, but not with H_3_-15 and H_3_-20, indicated that these protons (H-2, H-9, H-10, H-11 and H-12) were situated on the same face, and these were assigned as α protons, since the C-15 and C-20 methyls are β-substituents at C-1 and C-11, respectively. H-14 was found to exhibit an interaction with H_3_-15, but not with H-10, revealing the β-orientation of this proton. The configuration at C-9 is worthy of comment. H-9 was found to exhibit correlations with H-10, H-11, H_3_-18 and H_3_-20. From a consideration of molecular models, H-9 was found to be reasonably close to H-10, H-11, H_3_-18 and H_3_-20, while it was placed on the α face in **1**. The C-16 vinyl methyl showed correlations with H-4 and H-6, demonstrating the *Z* configuration of Δ^4,5^ and the hydroperoxy group at C-6 was α-oriented. The *cis* relationship between H-6 and H-7 was established by a correlation between H-6 and H-7 and a small coupling constant (*J* = 2.4 Hz) between these two protons. Moreover, an acetyl methyl (δ_H_ 2.01) exhibited correlations with H-12 and H-2, further supporting an acetoxy group was positioned on the α-position at C-14 in **1**. Based on the above findings, the configurations of all chiral carbons of **1** were assigned as 1S*, 2S*, 6S*, 7S*, 8R*, 9S*, 10S*, 11R*, 12S*, 14S*, 17R*, and the structure of **1** was established unambiguously. To the best of our knowledge, briarane derivatives possessing a hydroperoxy group are rarely found [22,32,38] and briarenolide F (**1**) is the first briarane derivative possessing a 6-hydroperoxy group. A double bond positioned at C-4(5) in briarane-type metabolites is also rarely found [31,40,41,42].

Briarenolide G (**2**) was isolated as a white powder whose HRESIMS showed a molecular ion at *m/z* 397.1989 implying that **2** had the molecular formula C_22_H_30_O_5_ (C_22_H_30_O_5_Na, calculated 397.1991). The IR spectrum revealed absorptions for hydroxy (3397 cm^−1^) and ester carbonyl (1757 and 1734 cm^−^^1^) groups. The ^1^H NMR data (Table 2) showed resonances due to an acetyl methyl (δ_H_ 2.03, 3H, s), three vinyl methyls (δ_H_ 1.78, 3H, br s, H_3_-16; 1.87, 3H, d, *J* = 1.6 Hz, H_3_-18; 1.63, 3H, d, *J* = 0.8 Hz, H_3_-20), a quaternary methyl (δ_H_ 0.77, 3H, s, H_3_-15), two olefinic protons (δ_H_ 5.29, 1H, br s, H-6; 5.12, 1H, m, H-12) and an oxymethine signal (δ_H_ 4.78, 1H, br s, H-14). The ^13^C NMR and DEPT spectra of **2** (Table 2) revealed the presence of a tetrasubstituted (δ_C_ 160.8, qC-8; 125.1, qC-17) and two trisubstituted (δ_C_ 144.4, qC-5; 124.6, CH-6; 136.3, qC-11; 117.8, CH-12) carbon-carbon double bonds, a hemiketal carbon (δ_C_ 106.7, qC-7), an acetate carbonyl (δ_C_ 170.9, qC), an α,β-unsaturated-γ-lactone carbonyl (δ_C_ 171.1, qC-19), a tetrasubstituted carbon atom bearing a carbon substituent (δ_C_ 39.1, qC-1) and an oxymethine (δ_C_ 77.2, CH-14).

**Table 2:** ^1^H (400 MHz, CDCl_3_) and ^13^C (100 MHz, CDCl_3_) NMR data, ^1^H–^1^H COSY and HMBC correlations for briarane **2**.

C/H	δ_Η_ (*J* in Hz)	δ_C_, Mult.	^1^H–^1^H COSY	HMBC (H→C)
1		39.1, qC		
2α	1.69 m	35.4, CH_2_	H-2β, H_2_-3	C-3, -14
β	1.29 m		H-2α, H_2_-3	C-1, -3, -10, -14
3	1.72 m	23.7, CH_2_	H_2_-2, H_2_-4	C-2
4α	1.90 m	29.6, CH_2_	H_2_-3, H-4β	N.O.
β	3.72 m		H_2_-3, H-4α	C-3, -16
5		144.4, qC		
6	5.29 br s	124.6, CH	H_3_-16	C-4, -7, -16
7		106.7, qC		
8		160.8, qC		
9α	2.54 br d (15.2)	25.3, CH_2_	H-9β, H-10	C-8, -10, -11, -17
β	2.37 dd (15.2, 10.8)		H-9α, H-10	C-7, -8, -10, -11, -17
10	3.76 d (10.8)	35.9, CH	H_2_-9	N.O.
11		136.3, qC		
12	5.12 m	117.8, CH	H_2_-13, H_3_-20	N.O.
13α	2.06 m	29.3, CH_2_	H-12, H-13β	C-11, -12, -14
β	2.33 m		H-12, H-13α	C-11
14	4.78 br s	77.2, CH	H_2_-13	C-12
15	0.77 s	21.9, CH_3_		C-1, -2, -10, -14
16	1.78 br s	23.9, CH_3_	H-6	C-4, -5, -6
17		125.1, qC		
18	1.87 d (1.6)	9.1, CH_3_		C-8, -17, -19
19		171.7, qC		
20	1.63 d (0.8)	21.9, CH_3_	H-12	C-10, -11, -12
7-OH	3.35 s			C-6, -7, -8
14-OAc		170.9, qC		
	2.03 s	21.7, CH_3_		Acetate carbonyl

N.O. = Not observed.

From the ^1^H–^1^H COSY experiment of **2** (Table 2), it was possible to establish the separate spin systems that map out the proton sequences from H_2_-2/H_2_-3/H_2_-4 and H_2_-9/H-10. These data, together with the HMBC correlations between H_2_-2/C-1, -3, -10; H_2_-3/C-2; H-4β/C-3; H-6/C-4, -7; and H_2_-9/C-7, -8, -10, established the connectivity from C-1 to C-10 in the ten-membered ring (Table 2). The vinyl methyl at C-5 was confirmed by the HMBC correlations between H_3_-16/C-4, -5, -6; H-4β/C-16; and H-6/C-16, and further supported by the allylic coupling between H-6 and H_3_-16. The methylcyclohexene ring, which is fused to the ten-membered ring at C-1 and C-10, was elucidated by the ^1^H–^1^H COSY correlations between H-12/H_2_-13/H-14 and H-12/H_3_-20 (by allylic coupling) and by the HMBC correlations between H_2_-2/C-14, H_2_-9/C-11 and H_3_-20/C-10, -11, -12. The ring junction C-15 methyl group was positioned at C-1 from the HMBC correlations between H_3_-15/C-1, -2, -10, -14. In addition, the acetate ester at C-14 was established by a correlation between H-14 (δ_H_ 4.78) and the acetate carbonyl observed in the HMBC spectrum of **2**. The presence of a hydroxy group at C-7 was deduced from the HMBC correlations between the hydroxy proton (δ_H_ 3.35, 1H, s, OH-7) and C-6, C-7, and C-8. The C-7 hydroxy group was concluded to be a part of hemiketal constellation on the basis of a characteristic carbon signal at δ_C_ 106.7 (a quaternary hemiketal carbon, qC-7). These data, together with the HMBC correlations between H_3_-18/C-8, -17, -19, were used to establish the molecular framework of **2**. 

**Figure 4:**
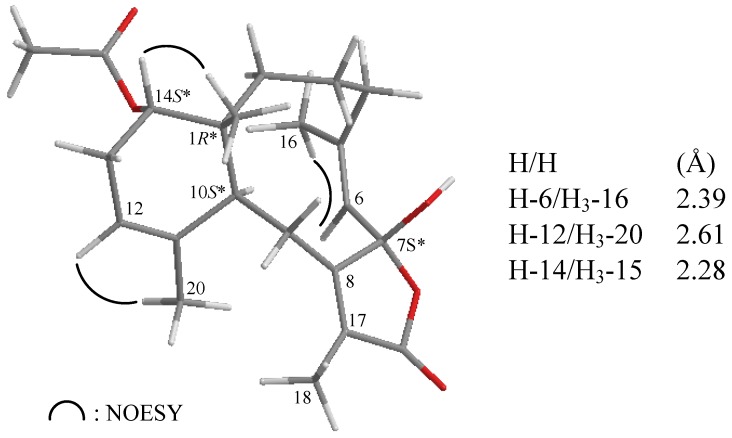
The stereoview of **2** (generated from computer modeling) and the calculated distances (Å) between selected protons with key NOESY correlations.

**Figure 5:**
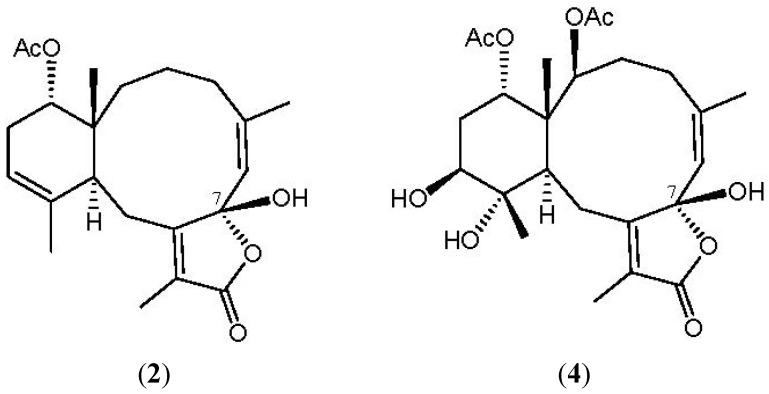
The structures of briarenolide G (**2**) and briarane (**4**).

NOESY measurements were carried out in order to deduce the relative stereochemical features of **2 **(Figure 4). Thus, H_3_-15 gave a correlation with H-14, but not with H-10, indicating that H_3_-15 and H-14 are located on the same face (assigned as the β-face) and that H-10 lies on the opposite side, α-face. The NOESY spectrum showed correlations between H-6/H_3_-16 and H-12/H_3_-20, revealing the *Z* geometry of the C-5/6 and C-11/12 double bonds in **2**. Due to the absence of NOESY correlations for the C-7 hydroxy group, the configuration at that chiral center could not be determined by this method. By comparison of the ^13^C NMR chemical shifts of C-6 (δ_C_ 124.6), C-7 (δ_C _106.7) and C-8 (δ_C_ 160.8) for **2** with those of an unnamed known 7β-hydroxybriarane analogue **4** (δ_C_ 124.8, C-6; 106.2, C-7, 160.1, C-8), which was obtained from a Caribbean octocoral *Briareum polyanthes* [43] (Figure 5), we deduced that the C-7 hydroxy group was β-oriented and the configuration of all the chiral carbons in **2** were assigned as 1R*, 7S*, 10S*, 14S*. 

The *in vitro* anti-inflammatory effects of briaranes **1** and **2** were tested. Briarenolide F (**1**) was found to display a significant inhibitory effect on the generation of superoxide anion by human neutrophils (Table 3).

**Table 3:** Inhibitory effects of briaranes **1** and **2 **on the generation of superoxide anion and the release of elastase by human neutrophils in response to FMLP/CB.

	Superoxide Anion		Elastase Release	
Compounds	IC_50_ (µg/mL)	Inh% *^a^*	IC_50_ (µg/mL)	Inh% *^a^*
**1**	3.82 ± 0.45	76.65 ± 4.21	>10.0	27.48 ± 6.60
**2**	>10.0	22.04 ± 3.43	>10.0	12.98 ± 4.68
DPI *^b^*	0.82 ± 0.31			
Elastatinal *^b^*			31.82 ± 5.92	

*^a^* Percentage of inhibition (Inh%) at a concentration of 10 µg/mL; *^b^* DPI (diphenylene indoniumn) and elastatinal were used as reference compounds.

## 3. Experimental Section

### 3.1. General Experimental Procedures

Optical rotations were measured on a Jasco P-1010 digital polarimeter. Infrared spectra were recorded on a Varian Diglab FTS 1000 FT-IR spectrometer; peaks are reported in cm^−1^. The NMR spectra were recorded on a Varian Mercury Plus 400 NMR spectrometer. Coupling constants (*J*) are given in Hz. ^1^H and ^13^C NMR assignments were supported by ^1^H–^1^H COSY, HMQC, HMBC and NOESY experiments. ESIMS and HRESIMS were recorded on a Bruker APEX II mass spectrometer. Column chromatography was performed on silica gel (230–400 mesh, Merck, Darmstadt, Germany). TLC was carried out on precoated Kieselgel 60 F_254_ (0.25 mm, Merck), and spots were visualized by spraying with 10% H_2_SO_4_ solution followed by heating. HPLC was performed using a system comprised of a Hitachi L-7100 pump and a Rheodyne injection port. A normal phase column (Hibar 250 × 10 mm, Merck, silica gel 60, 5 μm) was used for HPLC.

### 3.2. Animal Material

Specimens of the octocorals *Briareum* sp. were collected by hand using scuba equipment off the coast of southern Taiwan in July 2011 and stored in a freezer until extraction. A voucher specimen (NMMBA-TW-SC-2011-77) was deposited in the National Museum of Marine Biology and Aquarium. This organism was identified by comparison with previous descriptions [44,45,46,47]. 

### 3.3. Extraction and Isolation

Sliced bodies of *Briareum* sp. (wet weight 6.32 kg, dry weight 2.78 kg) were extracted with a mixture of methanol (MeOH) and dichloromethane (DCM) (1:1). The extract was partitioned between ethyl acetate (EtOAc) and H_2_O. The EtOAc layer was separated on silica gel and eluted using *n-*hexane/EtOAc (stepwise, 100:1–pure EtOAc) to yield 18 fractions A–R. Fraction H was chromatographed on silica gel and eluted using *n*-hexane/acetone (stepwise, 40:1–pure acetone) to afford 45 fractions H1–H45. Fraction H11 was separated by normal-phase HPLC (NP-HPLC) using a mixture of *n*-hexane and EtOAc (5:2) as the mobile phase to afford compound **2** (0.4 mg). Fraction H16 was further purified by normal-phase HPLC using a mixture of *n*-hexane and acetone as the mobile phase (7:2) to afford compound **1** (2.3 mg).

Briarenolide F (**1**): white powder; mp 141–142 °C; [α]25D +32 (*c* 0.1, CHCl_3_); IR (neat) ν_max_ 3498, 1789, 1743 cm^–1^; ^1^H (CDCl_3_, 400 MHz) and ^13^C (CDCl_3_, 100 MHz) NMR data, see Table 1; ESIMS: *m/z* 591 [M + Na]^+^; HRESIMS: *m/z* 591.2420 (calcd for C_28_H_40_O_12_Na, 591.2417).

Briarenolide G (**2**): white powder; mp 78–80 °C; [α]25D −97 (*c* 0.02, CHCl_3_); IR (neat) ν_max_ 3397, 1757, 1734 cm^–1^; ^1^H (CDCl_3_, 400 MHz) and ^13^C (CDCl_3_, 100 MHz) NMR data, see Table 2; ESIMS: *m/z* 397 [M + Na]^+^; HRESIMS: *m/z* 397.1989 (calcd for C_22_H_30_O_5_Na, 397.1991). 

### 3.4. Molecular Mechanics Calculations

Implementation of the MM2 force field [39] in CHEM3D PRO software from CambridgeSoft Corporation (version 9.0, Cambridge, MA, USA; 2005) was used to calculate the molecular models.

### 3.5. Superoxide Anion Generation and Elastase Release by Human Neutrophils

Human neutrophils were obtained by means of dextran sedimentation and Ficoll centrifugation. Measurements of superoxide anion generation and elastase release were carried out according to previously described procedures [48,49]. Briefly, superoxide anion production was assayed by monitoring the superoxide dismutase-inhibitable reduction of ferricytochrome *c*. Elastase release experiments were performed using MeO-Suc-Ala-Ala-Pro-Valp-nitroanilide as the elastase substrate.

## 4. Conclusions

Briarane-type natural products (3,8-cyclized cembranoid) were found in various marine organisms, particularly with the octocorals belonging to the genus *Briareum* (family Briareidae) [4,5,6,7]. It is interesting to note that the briarane-type natural products are major constituents of the extracts of octocorals *Briareum* spp. distributed in the tropical and subtropical Indo-Pacific Ocean. In the past 35 years, over 500 briarane analogues have been obtained and the number is still increasing based on their structural complexity and interesting bioactivities. It is worth noting that only three hydroperoxybriarane analogues have been isolated to date [22,32,38] and that briarenolide F (**1**) is the first 6-hydroperoxybriarane. 7-Hydroxybriarane derivatives are also rarely found [43,50,51,52]; the new briarane, briarenolide G (**2**) was the first 7-hydroxybriarane derivative isolated from the octocorals collected off the waters of Taiwan. The study material *Briareum* sp. has begun to be transplanted in tanks for the extraction of natural products in order to establish a stable supply of bioactive material.
